# Molecular epidemiology and antimicrobial resistance of *Haemophilus influenzae* in Guiyang, Guizhou, China

**DOI:** 10.3389/fpubh.2022.947051

**Published:** 2022-12-01

**Authors:** Yuhong Zhou, Yu Wang, Jinzhi Cheng, Xue Zhao, Yuedong Liang, Jiahong Wu

**Affiliations:** ^1^The Key Laboratory of Environmental Pollution Monitoring and Disease Control, Ministry of Education, School of Public Health, Guizhou Medical University, Guiyang, China; ^2^Department of Clinical Laboratory, The First People's Hospital of Guiyang, Guiyang, China; ^3^School of Basic Medical Sciences, Guizhou Medical University, Guiyang, China; ^4^Guiyang Public Health Treatment Center, Guiyang, China

**Keywords:** *Haemophilus influenzae*, antimicrobial resistance, molecular epidemiology, multilocus sequence type, *ftsI* gene

## Abstract

**Background:**

The widespread use of antimicrobials and *Haemophilus influenzae* type b (Hib) vaccine worldwide has altered the epidemiological patterns of invasive *H. influenzae*. Nonetheless, little is currently known on the epidemiological characteristics of *H. influenzae* in Guiyang, Guizhou, China.

**Objective:**

To determine the serotype distribution, antimicrobial resistance and Multilocus Sequence Typing (MLST) of *H. influenzae* in hospitalized patients in Guiyang City.

**Methods:**

A total of 196 clinical isolates from hospitalized patients were collected. Serotypes were determined according to the specific capsule gene, *bexA*, amplified by PCR. According to the guidelines of Clinical and Laboratory Standards Institute (CLSI) 2020 drug susceptibility tested, and the results determined. The chromogenic cephalosporin nitrocefin method was used to detect β-lactamase production, β-lactamase negative, ampicillin-resistant (BLNAR) strains were detected by PCR amplification and sequencing of the penicillin-binding protein 3 (PBP3) locus of *ftsI*. Multilocus Sequence Typing was performed for molecular typing.

**Results:**

All isolates studied were non-typeable *H. influenzae* (NTHi). Most patients originated from the pediatrics department (78.6%, 154/196), and suffered from lung with respiratory tract infection (pneumonia and bronchitis, 68.4%, 134/196). The resistance rates of ampicillin, cefaclor and azithromycin were 71.4% (140/196), 36.7% (72/196) and 34.2% (67/196), respectively. 40.3% (79/196) of strains were β-lactamase positive ampicillin-resistant (BLPAR). All BLPAR carried the TEM-1 gene. 9.2% (18/196) were β-lactamase negative ampicillin-resistant strains (BLNAR). The PBP3 mutation was detected in the ampicillin-resistant strains (*n* = 113), of which 18 belonged to group IIa. A total of 49 sequence types (ST) and 23 clonal complexes (CC) were detected, among which CC107 (ST107, *n* = 27; ST1002, *n* = 5; ST1218, *n* = 5) was the most frequent clonal complexes. BLPAR isolates mostly belonged to ST107 (20/79), while BLNAR was predominantly distributed in ST12 (5/18).

**Conclusion:**

*H. influenzae* infections are predominately caused by genetically diverse NTHi among hospitalized patients in Guiyang. The prevalence of β-lactamase production and PBP3 mutation may contribute to the high local ampicillin resistance rate.

## Introduction

*Haemophilus influenzae* (Hi) is a gram-negative bacterium that has been established to contribute to a wide variety of airway mucosal infections and invasive diseases such as bacterial meningitis. Before the introduction of Hib conjugate vaccines, Hib was the commonest cause of bacterial meningitis in young children in European countries (1) and is also reportedly the second most common bacterial pathogen causing pneumonia in Chinese children (2). Nowadays, *H. influenzae* strains are classified as typeable or non-typeable Hi based upon the presence of the polysaccharide capsule antigen, which also helps characterize typeable Hi into 6 serotypes (a to f), and lack of a capsule are identified as NTHi (non-typeable *H.influenzae*). Since the introduction of a conjugate vaccine against *H. influenzae* type b (Hib), invasive diseases of *H.influenzae* are now usually attributed to NTHi (3). In addition, *H.influenzae* infection is influenced by many factors, including the environment, population density and climatic conditions (4, 5). Accordingly, continuous monitoring of local and worldwide epidemiology is critical to help develop future disease control strategies.

Antibiotic resistance surveillance provides valuable information to aid clinicians in selecting empirical treatment. β-lactam antibiotics are nowadays commonly used in the clinical treatment of *H. influenzae*. In many countries, these empirical drugs have been found to be resistant (6). Rates of ampicillin resistant *H. influenzae* from <5% in several countries to 67.9% have been reported such as Canada, Japan, Italy, Shanghai and Taiwan (7). The resistance of *H. influenzae* to ampicillin is mainly through two mechanisms. One of the reported main resistance mechanisms involves the production of β-lactamase in *H.influenzae* that hydrolyze β-lactam antibiotics. Thus, strains that acquire β-lactamase genes such as TEM-1 and ROB-1 are referred to as β-lactamase-positive ampicillin-resistant *H. influenzae* (BLPAR) strains. The other mechanism involves β-lactamase-negative ampicillin-resistance (BLNAR), which arises from alterations of penicillin-binding protein 3 (PBP3) resulting from *ftsI* gene mutations, leading to the decreased affinity of β-lactamase antibiotics for PBP3 (7, 8). BLNAR isolates may represent a significant risk due to their ability to develop third-generation cephalosporin resistance (9).

Therefore, the classification of drug resistance phenotypes is important for clinical treatment and understanding the transmission of drug resistance genes. In this study, we aimed to preliminarily investigated the serotype distribution, antimicrobial resistance, and molecular epidemiology of *H. influenzae* among hospitalized patients in Guiyang, Guizhou, China.

## Materials and methods

### Samples

This investigation was conducted at *the Guiyang First People's Hospital*. A total of 196 isolates (only the first strain isolated from each patient was enrolled) were consecutively collected from the department of clinical microbiology, one central laboratory of this hospital, from June 2020 to July 2021. Respiratory tract specimens were collected for clinical examination according to the National Clinical Examination Procedures (4th edition), and immediately inoculated in chocolate agar, and incubated overnight at 35°C in 5% CO_2_. Only the first strain was included from each patient. Colonies resembling *H. influenzae* were identified by conventional methods, including requirement tests for hemin (X factor) and NAD (V factor). All preliminary identified *H.influenzae* isolates were further confirmed by testing for the *fucK* and *p6* genes, as previously described (10–12).

The studies involving human participants were reviewed and approved by Ethics Committee at *the Guiyang First People's Hospital* (Ethical approval No.G2020-S001).

### DNA templates preparation

An inoculated ring was selected and boiled in 200 μl of sterile distilled water for 10 min. Then the suspension was centrifuged at 12 000 rpm/min for 10 min. DNA was extracted quickly, and the supernatant was transferred to a second sterile tube and stored at −20°C until needed.

### Serotyping

Serotyping was performed by amplifying capsule-specific genes, *bexA*, using PCR as previously mentioned (13, 14). Strains that not possessed *bexA* gene were classified as NTHi.The details of primers sequences were indicated in Supplementary Table 1.

### Antimicrobial susceptibility test

The Kirby-Bauer disk-diffusion test was used to conduct drug sensitivity tests on *Haemophilus* test medium (HTM) plates and incubated for 24 h at 37°C with 5% CO_2_ in air, according to the Clinical and Laboratory Standards Institute (CLSI) (15). Commonly used antibacterial drugs in *H.influenzae* infected patients in the area were taken into consideration. Fourteen antibiotics were tested: ceftazidime, ampicillin, amoxicillin-clavulanic acid, meropenem, levofloxacin, imipenem, tetracycline, chloramphenicol, ampicillin-sulbactam, aztreonam, cefepime, rifampin, cefaclor, azithromycin. *H.influenzae* ATCC 49247 was used for quality control.

### β-lactamase and characterization of *ftsI* gene detection

All isolates were tested for the production of β-lactamase by the chromogenic cephalosporin nitrocefin method using known β-lactamase positives as controls. The *TEM-1* and *ROB-1* genes were identified by PCR as previously reported (16). The deduced amino acid sequence of the PBP3 transpeptidase region was aligned with the corresponding sequence from *H. influenzae* Rd KW20. The 140 ampicillin resistant strains were detected for *ftsI gene* and sequenced, and isolates with PBP3 mutation patterns were classified as previously reported (17).

### Multilocus sequence type

MLST genes (*atpG, frdB, pgi, adk, mdh, fucK and recA*) were amplified as described previously (18). Amplifications were performed in 50 μl total volumes of PCR reaction system contained ~10 μl of PreMix Taq (TaKaRa, Japan), 1 μl of forward and reverse primers, 2.5 μl of DNA, 35.5 μl of deionized water, respectively. Amplification was performed on an thermocycler using amplification parameters included an initial denaturation at 95°C for 4 min, followed by 30 cycles of 95°C for 30 s, 55°C for 30 s, 72°C for 60 s, then 72°C for 10 min. PCR products were detected by electrophoresis of 2 μl of each reaction on a 1.5% agarose gel for 30 min at 100 V, and the forward and reverse sequences were trimmed to the correct length and edited. The details of MLST primers sequences were indicated in Supplementary Table 1. All of the primers used were synthesized by Beijing-Tsingke Biotechnology Co., Ltd. (Beijing, China) with HPLC purification grade. In addition to providing clone descriptors, each unique allelic profile is assigned a sequence type (ST). ST with allelic variation at a single site (single locus variant, SLV), 2 sites (double locus variant, DLV) or 3 sites (triple locus variant, TLV) is considered to be a related part of the ST clonal complex (CC), while the difference in ST at more than 3 sites is considered to be unrelated. Allele numbers and ST were assigned by applying the *H.infuenzae* MLST website (https://pubmlst.org/hinfluenzae/) and analyzed and compared with the ST.

### Statistical analysis

The antimicrobial susceptibility data were analyzed with WHONET 5.6 software, as recommended by the World Health Organization. The chi-squared test and Fisher's exact test were used for statistical comparisons and performed with SPSS software. A two-tailed *P* < 0.5 was considered statistically significant. The MLST data of isolates of other provinces in China and worldwide isolates were come from the *H. influenzae* MLST website (www.pubmlst.org/hinfluenzae/). A UPGMA clustering analysis based on categorical coefficients was performed using the Bionumerics software package, version 4.0 (Applied Maths, Belgium) and a minimum spanning tree was constructed to determine phylogenetic pattern.

## Results

### Isolate distribution and patient characteristics

One hundred ninety-six clinical *H.influenzae* isolates (excluding strains from the same site of the same patient) were randomly selected from various respiratory tract specimens from children and adults hospitalized at *the Guiyang First People's Hospital* from June 2020 to July 2021, including sputum samples (*n* = 180), nasopharyngeal swabs (*n* = 15), and alveolar lavage fluid (*n* = 1) (Supplementary Table 2). The majority of specimens came from children (≤14 years old, *n* = 160) and the rest from adults (>14 years old, *n* = 36). Males (63.8%, 125/196) constituted a significant portion of patients compared to females (36.2%, 71/196). Based on the clinical diagnosis of hospitalized patients, lung with respiratory tract infection (pneumonia and bronchitis, 68.4%, 134/196) was the most common cause of infection (Supplementary Table 3). Most patients were from the pediatrics department (78.6%, 154/196), respiratory and critical care medicine department (6.1%, 12/196), and the otolaryngology department (3.1%, 6/196). The remaining cases were from the General department, Nephrology, and Gastroenterology department (Supplementary Table 4).

### Serotype and antimicrobial susceptibility

All isolates in this study were found to be NTHi. The drug resistance rates to ampicillin, cefaclor, azithromycin and amoxicillin-clavulanic acid were 71.4% (140/196), 36.7% (72/196), 34.2% (67/196), 26% (51/196), respectively, and high sensitivity to carbapenem, such as imipenem, meropenem and levofloxacin. The resistance rate of BLPAR to amoxicillin-clavulanic acid, ampicillin / sulbactam, Rifampin, Cefaclor was lower than that of BLNAR, but the resistance rate of Azithromycin was higher than that of BLNAR (*p* < 0.05) (Table 1). One multi-resistant *H. influenzae* isolate exhibited resistance to β-lactamase antimicrobias (ampicillin and amoxilin-clavulanate), carbapenem (imipenem, meropenem and levofloxacin), tetracycline, chloramphenicol and ampicillin-sulbactam (Table 2).

**Table 1:** Drug resistance rate and susceptibility rate of BLPAR and BLNAR strains to antibiotics.

**Antimicrobial**	**BLPAR**^a^ **(*****n*** = **79)**			**BLNAR**^b^ **(*****n*** = **18)**					**Total (*****n*** = **196)**	
	**Number of resistant strains**	Resistance rate (%)*	Sensitivity rate (%)*	**Number of resistant strains**	Resistance rate (%)*	Sensitivity rate (%)*	**χ^2^**	**P**	Resistance rate (%)^*^	Sensitivity rate (%)^*^
Ampicilin	79	100	0	18	100	0	–	–	71.4	22.9
Ceftazidime	3	3.8	91.1	1	5.6	94.4	0.000	>0.05	2	98
Amoxilin-clavulanate	0	0	100	18	100	0	90.496	< 0.05	26	73.5
Meropenem	0	0	100	1	5.6	94.4	0.661	>0.05	0.5	99.5
Levofloxacin	0	0	100	1	5.6	94.4	0.661	>0.05	0.5	99.5
Imipenem	0	0	100	1	5.6	94.4	0.661	>0.05	0.5	99.5
Tetracycline	20	25.3	60.8	5	27.8	44.4	0.102	>0.05	25.5	64.8
Chloramphenicol	7	8.9	70.9	1	5.6	61.1	0.000	>0.05	6.6	91.4
Ampicilin-sulbactam	12	15.2	82.3	17	94.4	5.6	42.782	< 0.05	32.7	65.3
Aztreonam	9	11.4	87.3	5	27.8	72.2	1.930	>0.05	13.3	86.2
Cefepime	0	0	100	0	0	100	–	–	0.5	99
Rifampin	8	10.1	65.8	6	33.3	50	4.001	< 0.05	13.8	72.9
Cefaclor	24	30.4	60.8	12	66.7	33.3	6.667	< 0.05	36.7	54.6
Azithromycin	48	60.8	38	1	5.6	94.4	18.342	< 0.05	34.2	65.3

a BLPAR: isolates which were β-lactamase positive, ampicillin resistant.

b BLNAR: isolates which were β-lactamase negative, ampicillin resistant.

* Some antimicrobial susceptibility rate add resistance rate < 100% is indicated in the table, mediating strain susceptibility to this antimicrobial agent.

**Table 2:** Multi-resistant *H. influenzae* isolate feature.

**Strains No**.	**ST**	**β-lactamase**	**PBP3 mutation**	**Antibiogram^a^**
262	12	–	No change	AMP-AMC-MEM-LEV-IPM-TCY-CHL-SAM

a AMP, ampicillin; AMC, amoxilin-clavulanate; MEM, meropenem; LEV, levofloxacin; IPM, imipenem; TCY, tetracycline; CHL, chloramphenicol; SAM, ampicillin-sulbactam.

### Production of β-lactamase and characterization of *ftsI* gene detection

The proportion of BLPAR and phenotypically BLNAR isolates was 40.3% (79/196) and 9.2% (18/196), respectively (Table 1). All 79 BLPAR isolates carried the *TEM-1* gene. One hundred thirteen ampicillin-resistant *H. influenzae* isolates had amino acid substitutions in PBP3. The isolates consisted of Group I (4.4%, 5/113), group IIa (15.9%, 18/113), group III (0.9%, 1/113), group III-like (41.6%, 47/113) (Table 3).

**Table 3:** Amino acid substitutions identified in the ftsI gene of *H. influenzae* isolates.

**Group**	**β-lactamase**	**n**	**Asp-350**	**Ser-357**	**Met-377**	**Ser-385**	**Leu-389**	**Gly-490**	**Ala-502**	**Arg-517**	**Asn-526**	**Ala-530**	**Thr-532**	**Val-547**	**Tyr-557**	**Gly-560**
I	+	3							Val	His						
	+	1							Val	His						Trp
	+	1								His						
IIa	+	9	Asn					Glu			Lys	Ser				
	+	5	Asn					Glu			Lys	Ser				Trp
	+	1	Asn					Glu			Lys	Ser			Lys	Trp
	-	1	Asn					Glu			Lys	Ser				Trp
	+	1									Lys					
	+	1									Lys	Ser				Trp
III	+	1	Asn	Asn	IIe	Thr				IIe	IIe	IIe	Leu	IIe	IIe	
	+	1	Asn	Asn	IIe	Thr	Phe				Lys	Ser				Trp
	+	2	Asn	Asn	IIe	Thr	Phe				Lys			IIe		Trp
	-	1	Asn	Asn	IIe	Thr	Phe	Glu			Lys	Ser			Asn	
	+	7	Asn	Asn	IIe	Thr	Phe	Glu			Lys	Ser				
III+IIa	-	2	Asn	Asn	IIe	Thr	Phe	Glu			Lys	Ser				
	-	2	Asn	Asn	IIe	Thr	Phe	Glu			Lys			IIe		
	+	3	Asn	Asn	IIe	Thr	Phe	Glu			Lys			IIe		
	+	2	Asn	Asn	IIe	Thr	Phe	Glu			Lys	Ser				Trp
	+	2	Asn	Asn	IIe	Thr	Phe				Lys			IIe	Asn	Val
	+	2	Asn	Asn	IIe	Thr	Phe	Glu			Lys	Ser				Trp
III+IIb	+	1	Asn	Asn	IIe	Thr	Phe	Glu	Val		Lys				Lys	
III+IIc	+	1	Asn	Asn	IIe	Thr	Phe		Thr		Lys					
	+	12	Asn	Asn	IIe	Thr	Phe			His			Ser	IIe	His	
	-	12	Asn	Asn	IIe	Thr	Phe			His			Ser	IIe	His	
	+	2	Asn	Asn	IIe	Thr	Phe			His					His	
	+	3	Asn	Asn	IIe	Thr	Phe			His			Ser	IIe		
III like	+	5	Asn	Asn	IIe	Thr	Phe			His			Ser	IIe	His	Trp
	-	3	Asn	Asn	IIe	Thr	Phe			His			Ser	IIe	His	Trp
	+	4	Asn	Asn	IIe	Thr	Phe			His				IIe	His	
	+	1	Asn	Asn	IIe	Thr				His			Ser	IIe	IIe	
	+	4	Asn	Asn	IIe	Thr				His			Ser	IIe		
	-	1	Asn	Asn	IIe	Thr				His			Ser	IIe		
Miscellaneous	+	2														Trp
	+	1												IIe		
	-	1												IIe		
	+	7	Asn		IIe				Val		Lys			IIe		
	+	3	Asn			Thr		Glu			Lys	Ser				Trp
	+	1	Asn	Asn							His					
	+	1	Asn	Asn		Thr	Phe	Glu				Ser	Ser	IIe		
No changes	+	21														
	-	6														
Total		140														

### MLST analysis of *H. influenzae*

**Figure 1:**
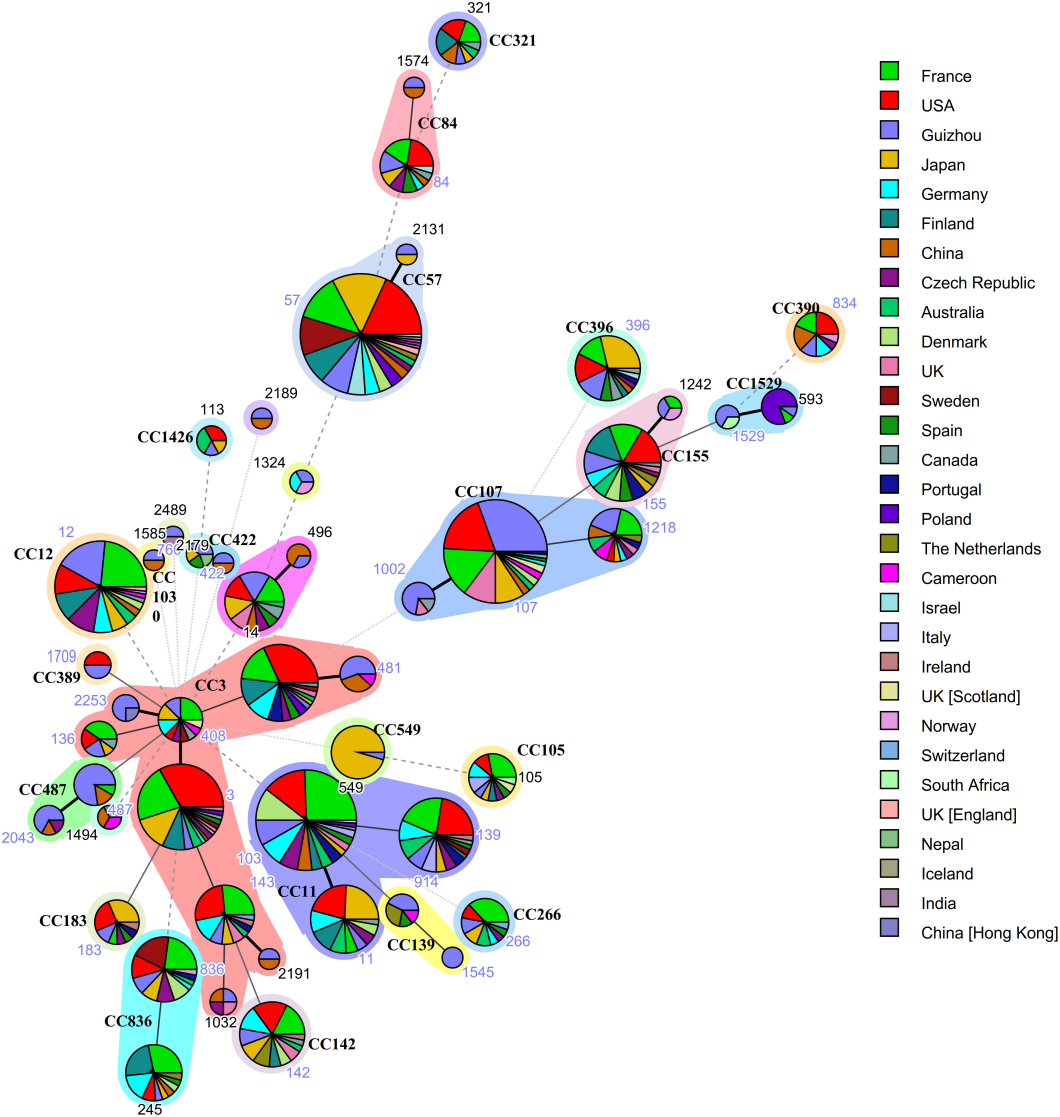
Genetic relationships based on minimum spanning tree of the 161 MLST profiles of *H. influenzae* isolates from Guizhou province and isolates of worldwide distribution. Each circle corresponds to a MLST profiles. The shadow zones in different color correspond to different clonal complexes. The size of the circle is proportional to the number of the isolates, and the color within the cycles represents the locality origin. Numbers in nodes denote sequence types and clonal complexes (CC), of which 49 different ST and 23 CC were identified.The solid and dotted lines indicate the single-locus variant and the double-locus variant, respectively.

Multilocus sequence typing was performed on all 196 isolates in our collection, and complete MLST data is available for 161 isolates. Of the 161 isolates that MLST data was available for, a total of 49 sequence types (ST) were detected, and constituted 23 clonal complexes (CC) and 8 singletons (Figure 1). The largest clonal complexes included the following: CC107 (ST107, *n* = 27; ST1002, *n* = 5; ST1218, *n* = 5), CC3 (ST3, *n* = 2; ST14, *n* = 1; ST136, *n* = 2; ST143, *n* = 2; ST408, *n* = 2; ST481, n=5; ST1032, *n* = 1; ST2191, *n* = 1; ST2253, *n* = 3) and CC487 (ST487, *n* = 10; ST2043, *n* = 4). The 49 ST in this study and the ST searched in MLST database, which includes from other provinces of China and 25 other countries (France, USA, Japan, Germany, Finland, Czech Republic, Australia, Denmark, UK, Sweden, Spain, Canada, Portugal, Poland, The Netherlands, Cameroon, Israel, Italy, Ireland, Norway, Switzerland, South Africa, Nepal, Iceland, India) were used for comparison. Guizhou Province accounts for the largest proportion of CC107, while CC3 with the most allelic variation contained isolates from both China (including Guizhou Province and other provinces of China) and other countries. BLPAR isolates mostly belonged to ST107 (20/79); BLNAR was predominantly distributed in ST12(5/18).

## Discussion

*H. influenzae* is a Gram-negative bacterium responsible for respiratory tract infections, especially in children (19). After the introduction of the Hib conjugate vaccine, the prevalence of invasive *H. influenzae* has declined, leading to a change in the epidemiology of *H. influenzae*. However, it should be borne in mind that Hib conjugate vaccines was promoted in the 1980's, Asia far behind its introduction in Europe and America, particularly in developing countries (20), which has led to large differences in the epidemiological patterns of *H. influenzae* around the world. All 196 Hi strains in this study were from respiratory specimens, sputum samples mainly from pediatric patients with pneumonia. Drug sensitivity test showed that resistance to ampicillin, cefaclor and azithromycin was 71.4, 36.7, and 34.2%, respectively. Our ampicillin and azithromycin resistance rates were relatively higher than those reported by the National Bacterial Resistance Monitoring Network 2014–2019 (21), while the resistance rates for the remaining antibiotics were relatively lower. Many factors, such as chronic disease, medications, regions or age, may possibly contribute to the diversity. The application of the Clinical and Laboratory Standards Institute (CLSI) and the European Committee on Antimicrobial Susceptibility Testing (EUCAST) guidelines also led to many discrepancies. At present, it remains difficult to establish a “gold standard” method for detecting ampicillin resistance and a consensus definition of β-lactam resistance in *H. influenzae* (22). This increase in resistance emphasizes the need for careful selection of empirical medication.

The detection ampicillin resistance was 71.4%, resistance of the BLNAR strain to ampicillin-sulbactam, rifampin and cefaclor was higher than for the BLPAR strain. In comparison, the resistance of the BLPAR strain to azithromycin was higher than for the BLNAR strain (*P* < 0.05) (Table 1). After the addition of the enzyme inhibitor sulbactam, a decrease in drug resistance strains was observed, indicating that ampicillin-resistant strains in this region were mainly enzyme-producing bacteria, and their resistance genes were *TEM-1* type, and no *ROB-1* type, consistent with findings of a study by Qin et al. (23). Previously, BLNAR isolates were thought scarce in China (6). However, in our study, 18 isolates of BLNAR were identified from 196 test isolates (9.2%). A former report in Shanghai, China, and Beijing, China, the detection rate of BLNAR strains was 11.76 and 18.9% (24, 25). In other countries, especially, Japan, BLNAR strains prevalence was >40% (3). The mechanism of BLNAR resistance involves the transposition of amino acid sites of the *ftsI* gene on the cell wall of penicillin-binding protein 3, which changes the spatial conformation. Importantly, three important mutations have been documented near the conservative motif: STVK (Ser-Thr-Val-Lys), SSN (Ser-Ser-Asn) and motif (Lys-Thr-Gly) (26). The amino acid site replacement of *ftsI* gene in isolates was 57.7% (113/196), and BLNAR were mostly distributed in the class III-like group (Table 3), which contributed to a 10–60-fold increase in MIC values of cefixime and cefuroxime, causing higher resistance than groups I and groups II (27). It has been shown that isolates with significant PBP3 mutations are more resistant to β-lactam antibiotics, resulting in decreased affinity between bacteria and β-lactam antibiotics. This phenomenon explains the unaltered antimicrobial activity in response to β-lactamase inhibitors and first and second-generation cephalosporins (28). BLNAR strains, which can potentially develop resistance to ampicillin, first- and second-generation cephalosporins in this region, can lead to high resistance rates, raising awareness on the need to strengthen the epidemic surveillance of BLNAR strains.

It has been established that multiple drug resistance (MDR) is the resistance of organisms to three or more different antimicrobials (29). In the present study, one multi-resistant *H. influenzae* isolate exhibited resistance to β-lactamase antimicrobias (ampicillin and amoxilin-clavulanate), carbapenem (imipenem, meropenem and levofloxacin), tetracycline, chloramphenicol and ampicillin-sulbactam (Table 2), which a number of studies in Japan and Thailand have shown a gradual increase in MDR Hi (30, 31). The situation of *H.influenzae* mechanisms of multiple drug resistance is not clear, which may result from the interplay of multiple factors, including the production of β-lactamase or *ftsI* gene mutations, plasmid exchange, and efflux pumps.

All 196 *H.influenzae* isolated in this study were NTHi, and cannot be prevented by Hib vaccine. MLST is widely acknowledged to identify pathogen strains by sequencing seven housekeeping genes; it can compare isolates from different laboratories since the allele profiles, and epidemiological information of strains can be stored in databases on the Internet. This approach has become increasingly popular to study clonal pathogen expansion (18). An increasing body of evidence suggests a high level of genetic diversity among the isolates, related to the genetic heterogeneity of the NTHi strains (32, 33). Guizhou Province accounts for the largest proportion of CC107 and ST107 was the most common sequence type in this study which is different from other provinces of China (Shanghai, Guangzhou, Beijing) (24, 25, 34) and other countries (Italy, Malaysia, Canada, Japan) (35–38). However, CC107 appears in multiple countries suggesting no geographical connection (Figure 1). This finding demonstrates the geographic heterogeneity of *H.influenzae* strains that have evolved independently. Despite the genetic heterogeneity of the NTHi isolates, we observed an association between ampicillin resistance status and ST. In this regard, β-lactamase has been established to be mainly carried by ST107 and ST103, while some BLNAR isolates belong to ST12. ST103 and ST14 have been reported in Italy, Portugal, Ireland and are associated with invasive disease (39–41). ST14 has recently been documented in Sweden and is thought to be associated with increased virulence and persistence (42, 43) and which was included the most common CC3. The presence of ST103 and ST14 with loci mutations underscores the importance of surveillance studies, including molecular typing of these isolates.

Some limitations were noted in our study. First of all, the vaccination data of enrolled patients was not available. Moreover, PCR detection of pathogens was not carried out in patients with cold-like symptoms whose sputum culture was negative. Finally, our sampling was biased toward non-invasive bacterial isolates, as sputum samples were predominantly collected while other specimens such as blood were not. However, to the best of our knowledge, this is the first systematic investigation of *H. influenzae* colonization in the Guiyang area. Importantly, our study provides important information on potential high-risk genotypes to be tracked during epidemiological surveillance.

## Conclusion

In conclusion, although data on the distribution of serotypes carrying *H.influenzae* isolates in our region prior to vaccination is unavailable, it is now clear that NTHi is overwhelmingly dominant and cannot be prevented by Hib vaccination. PBP3 mutations and β-lactamase production are prevalent in *H. influenzae* strains, leading to ampicillin resistance and reduced sensitivity to other β-lactamase antibiotics. Despite the high genetic diversity of NTHi, we found an association between ampicillin resistance status and specific clonal complexes; specific genotypes may have a higher potential for aggressive disease. To our knowledge, this is the first report of NTHi sequence typing in Guiyang. Indeed, monitoring NTHi colonization rates and prevailing genotypes will provide useful information for better understanding the evolution of *H. influenzae* disease and assist in vaccine development.

## Data availability statement

The original contributions presented in the study are included in the article/Supplementary material, further inquiries can be directed to the corresponding authors.

## Ethics statement

The study was approved by the Human Ethics Committee of the First People's Hospital of Guiyang (Approval No.No.G2020-S001) and complied with the Declaration of Helsinki. Before our team obtained the samples/isolates and conducted the study, any personal identifiers of the suspected *H. influenzae*-infected patients had been removed by the monitoring stations. So, the patient informed consent was waived by the Human Ethics Committee of the First People's Hospital of Guiyang.

## Author contributions

YW, YZ, and JW conceived and designed the experiments and wrote the paper. YZ, XZ, and JC performed the experiments. YZ and YW analyzed the data. All authors contributed to the article and approved the submitted version.

## Funding

This study was supported by [2019] Zhu wei jian ke ji he tong zi di 001, Zhu ke he tong [2020]-10-6 and Zhu ke he tong [2021]-43-25 from Science and Technology Department of Guiyang city of Guizhou Province, and was supported by Qian Ke He Zhi Cheng [2021] Yi Ban 440 from Science and Technology Department of Guizhou Province.

## Conflict of interest

The authors declare that the research was conducted in the absence of any commercial or financial relationships that could be construed as a potential conflict of interest.

## Publisher's note

All claims expressed in this article are solely those of the authors and do not necessarily represent those of their affiliated organizations, or those of the publisher, the editors and the reviewers. Any product that may be evaluated in this article, or claim that may be made by its manufacturer, is not guaranteed or endorsed by the publisher.

## References

[1] Slack MPE. Long term impact of conjugate vaccines on *Haemophilus influenzae* meningitis: narrative review. Microorganisms. (2021) 9:886. 10.3390/microorganisms9050886 33919149 PMC8143157

[2] Zhu H Wang A Tong J Yuan L Gao W Shi W. Nasopharyngeal carriage and antimicrobial susceptibility of *Haemophilus influenzae* among children younger than 5 years of age in Beijing, China. BMC Microbiol. (2015) 15:6. 10.1186/s12866-015-0350-7 25648185 PMC4332420

[3] Miyahara R Suzuki M Morimoto K Chang B Yoshida S Yoshinaga S. Nosocomial outbreak of upper respiratory tract infection with beta-lactamase-negative ampicillin-resistant nontypeable *Haemophilus influenzae*. Infect Control Hosp Epidemiol. (2018) 39:652–9. 10.1017/ice.2018.56 29611493

[4] Brown NE Blain AE Burzlaff K Harrison LH Petit S Schaffner W. Racial disparities in invasive *Haemophilus influenzae* disease—United States, 2008–2017. Clin Infect Dis. (2021) 73:1617–24. 10.1093/cid/ciab449 33993217 PMC11307574

[5] Mazamay S Bompangue D Guégan JF Muyembe JJ Raoul F Broutin H. Understanding the spatio-temporal dynamics of meningitis epidemics outside the belt: the case of the Democratic Republic of Congo (DRC). BMC Infect Dis. (2020) 20:291. 10.1186/s12879-020-04996-7 32312246 PMC7168871

[6] Guitor AK Wright GD. Antimicrobial resistance and respiratory infections. Chest. (2018) 154:1202–12. 10.1016/j.chest.2018.06.019 29959904

[7] Wen S Feng D Chen D Yang L Xu Z. Molecular epidemiology and evolution of *Haemophilus influenzae*. Infect Genet Evol. (2020) 80:104205. 10.1016/j.meegid.2020.104205 31981610

[8] Dabernat H Delmas C Seguy M Pelissier R Faucon G Bennamani S. Diversity of beta-lactam resistance-conferring amino acid substitutions in penicillin-binding protein 3 of *Haemophilus influenzae*. Antimicrob Agents Chemother. (2002) 46:2208–18. 10.1128/AAC.46.7.2208-2218.2002 12069976 PMC127296

[9] Deghmane AE Hong E Chehboub S Terrade A Falguieres M Sort M. High diversity of invasive *Haemophilus influenzae* isolates in France and the emergence of resistance to third generation cephalosporins by alteration of ftsI gene. J Infect. (2019) 79:7–14. 10.1016/j.jinf.2019.05.007 31100360

[10] Tian GZ Zhang LJ Wang XL Zhang L Li SF Gu CM. Rapid detection of *Haemophilus influenzae* and *Haemophilus parainfluenzae* in nasopharyngeal swabs by multiplex PCR. Biomed Environ Sci. (2012) 25:367–71. 10.3967/0895-3988.2012.03.016 22840589

[11] van Ketel RJ de Wever B van Alphen L. Detection of *Haemophilus influenzae* in cerebrospinal fluids by polymerase chain amplification DNA amplification. J Med Microbiol. (1990):33:271–6. 10.1099/00222615-33-4-271 2258914

[12] Abdeldaim GM Stralin K Olcen P Blomberg J Molling P Herrmann B. Quantitative fucK gene polymerase chain reaction on sputum and nasopharyngeal secretions to detect *Haemophilus influenzae* pneumonia. Diagn Microbiol Infect Dis. (2013) 76:141–6. 10.1016/j.diagmicrobio.2013.02.015 23541117

[13] Davis GS Sandstedt SA Patel M Marrs CF Gilsdorf JR. Use of bexB to detect the capsule locus in *Haemophilus influenzae*. J Clin Microbiol. (2011) 49:2594–601. 10.1128/JCM.02509-10 21525217 PMC3147886

[14] Falla TJ Crook DW Brophy LN Maskell D Kroll JS Moxon ER. For capsular typing of Haemophilus influenzae. J Clin Microbiol. (1994) 32:2382–6. 10.1128/jcm.32.10.2382-2386.1994 7814470 PMC264070

[15] CLSI. Methods for Dilution Antimicrobial Susceptibility Tests for Bacteria That Grow Aerobically; Approved Standard—Tenth Edition. CLSI document M100. Wayne, PA: Clinical and Laboratory Standards Institute (2020).

[16] Luo C Xia Y Liu Q Chu L Fu X Jing C. Antibiotic resistance and molecular epidemiology of the beta-lactamase-producing *Haemophilus influenzae* isolated in Chongqing, China. APMIS. (2012) 120:926–34. 10.1111/j.1600-0463.2012.02921.x 23009117

[17] Garcia-Cobos S Campos J Lazaro E Roman F Cercenado E Garcia-Rey C. Ampicillin-resistant non-beta-lactamase-producing *Haemophilus influenzae* in Spain: recent emergence of clonal isolates with increased resistance to cefotaxime and cefixime. Antimicrob Agents Chemother. (2007) 51:2564–73. 10.1128/AAC.00354-07 17470649 PMC1913223

[18] Meats E Feil EJ Stringer S Cody AJ Goldstein R Kroll JS. Characterization of encapsulated and noncapsulated *Haemophilus influenzae* and determination of phylogenetic relationships by multilocus sequence typing. J Clin Microbiol. (2003) 41:1623–36. 10.1128/JCM.41.4.1623-1636.2003 12682154 PMC153921

[19] Kehl SC Dowzicky MJ. Global assessment of antimicrobial susceptibility among gram-negative organisms collected from pediatric patients between 2004 and 2012: results from the tigecycline evaluation and surveillance trial. J Clin Microbiol. (2015) 53:1286–93. 10.1128/JCM.03184-14 25653413 PMC4365249

[20] Adams WG Deaver KA Cochi SL Plikaytis BD Zell ER Broome CV. Decline of childhood *Haemophilus influenzae* type b (Hib) disease in the Hib vaccine era. JAMA. (1993) 269:221–6. 10.1001/jama.269.2.221 8417239

[21] Network TNBDRM. National bacterial resistance monitoring network for 2014-2019. Chin J Anim Infect Dis. (2021) 20:15–31. 10.12138/j.issn.1671-9638.20216170

[22] Kosikowska U Andrzejczuk S Grywalska E Chwiejczak E Winiarczyk S Pietras-Ozga D. Prevalence of susceptibility patterns of opportunistic bacteria in line with CLSI or EUCAST among *Haemophilus parainfluenzae* isolated from respiratory microbiota. Sci Rep. (2020) 10:11512. 10.1038/s41598-020-68161-5 32661300 PMC7359364

[23] Qin HH Pan F Liu CQ Zhang H. Isolation of drug resistance and beta-lactamase genotyping of *H. influenzae* in children. J Clin InspecT. (2019) 37:48–50. 10.13602/j.cnki.jcls.2019.01.12

[24] Li XX Xiao SZ Gu FF He WP Ni YX Han LZ. Molecular epidemiology and antimicrobial resistance of *Haemophilus influenza*e in adult patients in Shanghai, China. Front Public Health. (2020) 8:95. 10.3389/fpubh.2020.00095 32292774 PMC7135888

[25] Dong Q Shi W Cheng X Chen C Meng Q Yao K. Widespread of non-typeable *Haemophilus influenzae* with high genetic diversity after two decades use of Hib vaccine in China. J Clin Lab Anal. (2020) 34:e23145. 10.1002/jcla.23145 31846125 PMC7171301

[26] Osaki Y Sanbongi Y Ishikawa M Kataoka H Suzuki T Maeda K. Genetic approach to study the relationship between penicillin-binding protein 3 mutations and *Haemophilus influenzae* beta-lactam resistance by using site-directed mutagenesis and gene recombinants. Antimicrob Agents Chemother. (2005) 49:2834–9. 10.1128/AAC.49.7.2834-2839.2005 15980357 PMC1168665

[27] Kitaoka K Kimura K Kitanaka H Banno H Jin W Wachino JI. Carbapenem-nonsusceptible *Haemophilus influenzae* with penicillin-binding protein 3 containing an amino acid insertion. Antimicrob Agents Chemother. (2018) 62:e00671-18. 10.1128/AAC.00671-18 29784853 PMC6105787

[28] Straker K Wootton M Simm AM Bennett PM MacGowan AP Walsh TR. Cefuroxime resistance in non-beta-lactamase *Haemophilus influenzae* is linked to mutations in ftsI. J Antimicrob Chemother. (2003) 51:523–30. 10.1093/jac/dkg107 12615852

[29] Magiorakos AP Srinivasan A Carey RB Carmeli Y Falagas ME Giske CG. Multidrug-resistant, extensively drug-resistant and pandrug-resistant bacteria: an international expert proposal for interim standard definitions for acquired resistance. Clin Microbiol Infect. (2012) 18:268–81. 10.1111/j.1469-0691.2011.03570.x 21793988

[30] Yamada S Seyama S Wajima T Yuzawa Y Saito M Tanaka E. β-Lactamase-non-producing ampicillin resistant *Haemophilus influenzae* is acquiring multidrug resistance. J Infect Public Health. (2020) 13:497–501. 10.1016/j.jiph.2019.11.003 31839585

[31] Tribuddharat C Srifuengfung S. Multiple drug resistance in *Haemophilus influenzae* isolated from patients in Bangkok, Thailand. J Glob Antimicrob Resist. (2017) 9:121–3. 10.1016/j.jgar.2017.03.003 28506825

[32] Erwin AL Sandstedt SA Bonthuis PJ Geelhood JL Nelson KL Unrath WC. Analysis of genetic relatedness of *Haemophilus influenzae* isolates by multilocus sequence typing. J Bacteriol. (2008) 190:1473–83. 10.1128/JB.01207-07 18065541 PMC2238191

[33] Giufrè M Cardines R Accogli M Pardini M Cerquetti M. Identification of *Haemophilus influenzae* clones associated with invasive disease a decade after introduction of *H. influenzae* Serotype b Vaccination in Italy. Clin Vaccine Immunol. (2013) 20:1223–9. 10.1128/CVI.00028-13 23761663 PMC3754497

[34] Chen D Wen S Feng D Xu R Liu J Peters BM. Microbial virulence, molecular epidemiology and pathogenic factors of fluoroquinolone-resistant *Haemophilus influenzae* infections in Guangzhou, China. Ann Clin Microbiol Antimicrob. (2018) 17:41. 10.1186/s12941-018-0290-9 30470228 PMC6251178

[35] Giufre M Daprai L Cardines R Bernaschi P Rava L Accogli M. Carriage of *Haemophilus influenzae* in the oropharynx of young children and molecular epidemiology of the isolates after fifteen years of *H. influenzae* type b vaccination in Italy. Vaccine. (2015) 33:6227–34. 10.1016/j.vaccine.2015.09.082 26440924

[36] Mohd-Zain Z Kamsani NH Ahmad N Clarke SC. Phylogenetic relationship of non-typeable *Haemophilus influenzae* isolated in Malaysia. Infect Genet Evol. (2015) 36:240–3. 10.1016/j.meegid.2015.09.017 26394107

[37] Tsang RSW Shuel M Ahmad T Hayden K Knox N Van Domselaar G. Whole genome sequencing to study the phylogenetic structure of serotype a *Haemophilus influenzae* recovered from patients in Canada. Can J Microbiol. (2020) 66:99–110. 10.1139/cjm-2019-0406 31661630

[38] Adachi Y Ando M Morozumi M Ubukata K Iwata S. Genotypic characterization of *Haemophilus influenzae* isolates from paediatric patients in Japan. J Med Microbiol. (2018) 67:695–701. 10.1099/jmm.0.000721 29595417

[39] Heliodoro CIM Bettencourt CR Bajanca-Lavado MP; Portuguese Group for the Study of Haemophilus influenzae invasive infection. Molecular epidemiology of invasive Haemophilus influenzae disease in Portugal: an update of the post-vaccine period, 2011-2018. Eur J Clin Microbiol Infect Dis. (2020) 39:1471–80. 10.1007/s10096-020-03865-0 32172370

[40] Giufre M Fabiani M Cardines R Riccardo F Caporali MG D'Ancona F. Increasing trend in invasive non-typeable *Haemophilus influenzae* disease and molecular characterization of the isolates, Italy, 2012-2016. Vaccine. (2018) 36:6615–22. 10.1016/j.vaccine.2018.09.060 30292458

[41] McElligott M Meyler K Bennett D Mulhall R Drew RJ Cunney R. Epidemiology of *Haemophilus influenzae* in the Republic of Ireland, 2010-2018. Eur J Clin Microbiol Infect Dis. (2020) 39:2335–44. 10.1007/s10096-020-03971-z 32666480

[42] Andersson M Resman F Eitrem R Drobni P Riesbeck K Kahlmeter G. Outbreak of a beta-lactam resistant non-typeable *Haemophilus influenzae* sequence type 14 associated with severe clinical outcomes. BMC Infect Dis. (2015) 15:581. 10.1186/s12879-015-1319-8 26700635 PMC4690285

[43] Månsson V Skaare D Riesbeck K Resman F. The spread and clinical impact of ST14CC-PBP3 type IIb/A, a clonal group of non-typeable *Haemophilus influenzae* with chromosomally mediated β-lactam resistance—a prospective observational study. Clin Microbiol Infect. (2017) 23: 209.e1–7. 10.1016/j.cmi.2016.11.006 27852000

