# Supplementary material for: Molecular epidemiology and antimicrobial resistance of Haemophilus influenzae in Guiyang, Guizhou, China
Source: Front Public Health. 2022 Dec 1;10:947051. doi: 10.3389/fpubh.2022.947051 (PMC9751421; doi:10.3389/fpubh.2022.947051)
Supplement: Supplementary file 1 [file Table_1.DOCX]

**Supplementary Table 1** Primers used in this study for analysis of *H.influenzae*

| gene | Length of sequenced fragment(bp) | Sequences( from 5' end to 3' end) | Annealing temperature（℃） | |
| --- | --- | --- | --- | --- |
| P6 | 250 | F:TTGGCGGTTACTCTGTTGCT | 50 | |
|  |  | R:TGCAGGTTTTTCTTCACCGT |  |  |
| fucK | 550 | F:ACCACTTTCGGCGTGGATGG | 55 |  |
|  |  | R:AAGATTTCCCCCAGGTGCCAGA |  |  |
| bexA | 343 | F:CGTTTGTATGATGTTGATCCAGAC | 50 |  |
|  |  | R:TGTCCATGTCTTCAAAATGATG |  |  |
| TEM-1 | 400 | F:ATCAGTTGGGTGCACGAGTG | 56 | |
|  |  | R:CGCTCGTCGTTTGGTATGG |  |  |
| ROB-1 | 338 | F:ACCCATACCAATCGGCTAGA | 52 | |
|  |  | R:TGGCGTCTTCGGTAAATTGC |  |  |
| ftsI | 1050 | F:AGCTGCTTCAGCATCTTGC | 52 | |
|  |  | R:TTGCACATATCTCCGATGAG |  |  |
| adk | 477 | F:GGTGCACCGGGTGCAGGTAA  R:CCTAAGAT TTTATCTAACTC | 55 | |
| atpG | 447 | F:ATGGCAGGTGCAAAAGAGAT  R:TTGTACAACAGGCTTTTGCG |  |  |
| frdB | 489 | F:CTTATCGTTGGTCTTGCCGT  R:TTGGCACTTTCCACTTTTCC |  |  |
| fucK | 345 | F:ACCACTTTCGGCGTGGATGG  R:AAGATTTCCCAGGTGCCAGA |  |  |
| mdh | 405 | F:TCATTGTATGATATTGCCCC  R:ACTTCTGTACCTGCATTTTG |  |  |
| pgi | 468 | F:GGTGAAAAAATCAATCGTAC  R:ATTGAAAGACCAATAGCTGA |  |  |
| recA | 426 | F:ATGGCAACTCAAGAAGAAAA  R:TTACCAAACATCACGCCTAT |  |  |
