# Supplementary material for: Molecular epidemiology and antimicrobial resistance of Haemophilus influenzae in Guiyang, Guizhou, China
Source: Front Public Health. 2022 Dec 1;10:947051. doi: 10.3389/fpubh.2022.947051 (PMC9751421; doi:10.3389/fpubh.2022.947051)
Supplement: Supplementary file 2 [file Table_2.DOCX]

**Supplementary Table 2** Information of the samples used in this study

| Specimen type | amount |
| --- | --- |
| Sputum | 180 |
| Nasopharyngeal swabs | 15 |
| Alveolar lavage fluid | 1 |
| total | 196 |
