# Supplementary material for: Molecular epidemiology and antimicrobial resistance of Haemophilus influenzae in Guiyang, Guizhou, China
Source: Front Public Health. 2022 Dec 1;10:947051. doi: 10.3389/fpubh.2022.947051 (PMC9751421; doi:10.3389/fpubh.2022.947051)
Supplement: Supplementary file 3 [file Table_3.DOCX]

**Supplementary Table 3** Isolation of Haemophilus influenzae from different diseases

| diseases | number | separation rate(%) |
| --- | --- | --- |
| pneumonia | 93 | 47.4 |
| bronchitis | 41 | 20.9 |
| asthma | 2 | 1.0 |
| chronic obstructive pulmonary disease | 7 | 3.6 |
| amygdalitis | 26 | 13.3 |
| others^a^ | 27 | 13.8 |
| total | 196 | 100 |

^a^*Others were distributed in the diabetes, chronic kidney failure, hypertension etc.*
