# Supplementary material for: Molecular epidemiology and antimicrobial resistance of Haemophilus influenzae in Guiyang, Guizhou, China
Source: Front Public Health. 2022 Dec 1;10:947051. doi: 10.3389/fpubh.2022.947051 (PMC9751421; doi:10.3389/fpubh.2022.947051)
Supplement: Supplementary file 4 [file Table_4.DOCX]

**Supplementary Table 4** All patients hospitalized in clinical departments

| Department | number | rate(%) |
| --- | --- | --- |
| Pediatrics department | 154 | 78.6 |
| Respiratory and Critical care medicine department | 12 | 6.1 |
| Otolaryngology department | 6 | 3 |
| others^a^ | 24 | 12.2 |
| total | 196 | 100 |

*^a^Others were distributed in the department of General department, Nephrology department, Gastroenterology department, etc.*
